# Slit mesh technique in laparoscopic inguinal hernia repair: a retrospective analysis

**DOI:** 10.20452/wiitm.2025.17958

**Published:** 2025-06-06

**Authors:** Ozan M. Aydin, Yasin Kara, Serhan Yilmaz, Erkan Somuncu, Osman Sibic

**Affiliations:** Ministry of Health, General Surgery Service, Malazgirt State Hospital, Mus, Turkey; General Surgery Department, Kanuni Sultan Süleyman Training and Research Hospital, Istanbul, Turkey; Ministry of Health, General Surgery Department, Ankara Bilkent City Hospital, Ankara, Turkey; Ministry of Health, General Surgery Department, Derik State Hospital, Mardin, Turkey

**Keywords:** groin hernia, laparoscopic inguinal hernia repair, slit mesh, total extraperitoneal repair

## Abstract

**INTRODUCTION:**

Slit mesh (SM) is a technical modification used in laparoscopic total extraperitoneal (TEP) inguinal hernia (IH) repair. It aims to reduce recurrence by improving mesh anchoring and preventing cranial migration. However, its clinical effectiveness remains controversial.

**AIM:**

The aim of this study was to compare clinical outcomes of SM and nonslit mesh (NSM) techniques in laparoscopic TEP IH repair.

**MATERIALS AND METHODS:**

This retrospective study included 155 patients who underwent standardized TEP repair between June 2022 and February 2023. The patients were divided into 2 groups: SM (n = 80) and NSM (n = 75). Demographics, hernia characteristics, operative time, recurrence, complications, and postoperative pain were evaluated. Pain was assessed using the visual analogue scale (VAS) on postoperative day 1 (VAS1D), at 1 month (VAS1M), and 3 months (VAS3M).

**RESULTS:**

No significant differences were found in baseline characteristics. Median (interquaritle range [IQR]) operation time was longer in the SM group (46.5 [40–55] vs 38 [30–45] min; P <⁠0.001). Recurrence was observed in 6 SM and 3 NSM patients (odss ratio, 1.95; 95% CI, 0.47–8.08; P = 0.497). Median (IQR) VAS scores were: 4 (2–6) for VAS1D; 0 (0–1) for VAS1M; 0 (0–0) for VAS3M, with no significant differences. Complication and chronic postoperative inguinal pain rates were similar.

**CONCLUSIONS:**

Although the SM technique was designed to improve outcomes, our findings show no it has no notable advantage over the NSM technique in reducing recurrence or postoperative pain. Moreover, the prolonged operation time associated with the SM method may represent a clinical drawback. Further research with larger cohorts and longer follow-up is needed to better clarify the potential risks and benefits of SM apporoach.

## INTRODUCTION

Inguinal hernia (IH) repair is one of the most commonly performed surgical procedures in clinical practice, with approximately 15 million operations conducted annually. The lifetime risk of IH is estimated at 27%–43% in men and 3%–6% in women.^1^ Most cases are symptomatic and surgery remains the most definitive and effective treatment option. The primary goal of laparoscopic IH repair is to minimize recurrence and reduce the incidence of chronic postoperative inguinal pain (CPIP). Recurrence rates are reported to range from 2% to 17%.^1^ CPIP, defined as pain persisting longer than 3 months, occurs in approximately 18% of open and 6% of laparoscopic repairs.^1^**^,^**^2^

The slit mesh (SM) technique is a technical modification aimed at addressing these challenges. In a standard total extraperitoneal (TEP) repair, physical movement may stretch the spermatic cord and elevate the lower edge of the mesh, contributing to the pathophysiology of recurrence. Routing the mesh beneath the cord creates an additional anchoring point and improves defect coverage, thereby limiting mesh displacement and potential recurrence. However, current literature remains inconclusive regarding the superiority of the SM technique. While some studies^3^**^,^**^4^ suggest that it may help reduce recurrence, others^5^^,^^6^^,^^7^^,^^8^^,^^9^^,^^10^^,^^11^ raise concerns that creating an opening in the mesh might weaken its structural integrity. This ongoing debate underscores the need for further, well-designed studies to clarify the clinical implications of this technique.

**FIGURE 1:**
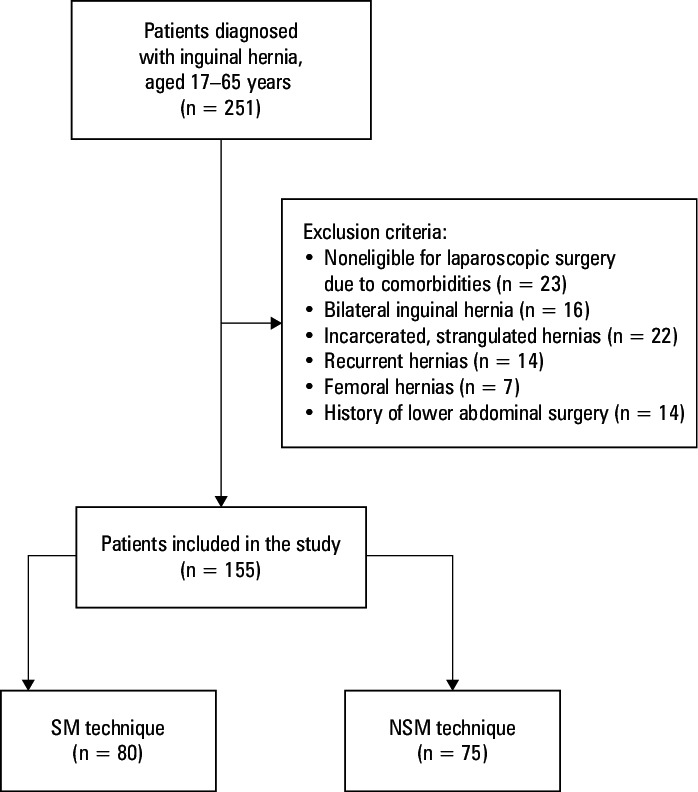
Study inclusion and exclusion criteria

Abbreviations: NSM, nonslit mesh; SM, slit mesh

## AIM

The aim of this study was to investigate the potential impact of SM placement on clinical outcomes in comparison with the NSM technique.

## MATERIALS AND METHODS

The study included patients diagnosed with unilateral IH and treated surgically at a tertiary training and research hospital between June 2022 and February 2023. The inclusion and exclusion criteria are presented in FIGURE 1 . This retrospective study was approved by the Clinical Research Ethics Committee of Kanuni Sultan Suleyman Training and Research Hospital (2022.07.172) and conducted in accordance with the Declaration of Helsinki. Written informed consent was obtained from all patients.

The patients were divided into 2 groups: SM and NSM. As this was a retrospective study, the choice of mesh type was solely based on the surgeon’s preference. The decision was made preoperatively and was not influenced by intraoperative findings, such as hernia type, size, or laterality. However, the possibility of selection bias cannot be entirely excluded. Both groups underwent myopectineal orifice dissection, and the surgical technique was identical up to this stage. In the SM group, an additional inferior dissection of the spermatic cord was performed to create a passage for the lateral leg of the mesh. In contrast, no further dissection was made in the NSM group.

The operations were carried out by 3 different surgeons with considerable experience in TEP procedure. According to the 2018 European Hernia Society (EHS) guidelines,^2^ such experience is defined as having performed at least 50–100 procedures. The patients were followed for approximately 12 months, with individual consultations conducted on postoperative day 1 and at 1 month, and telephone follow-ups at 3 and 12 months.

Preoperative data included age, sex, body mass index, and hernia defect diameter measured on ultrasonography. Based on ultrasonographic measurements, hernias were also classified according to the EHS system as class 1 (≤1.5 cm), class 2 (1.5–3 cm), or class 3 (>3 cm), and these classifications were used for further analysis. Intraoperative variables, such as the side and type of hernia, operation time, need for drainage, and need for conversion were recorded. During postoperative follow-up, length of hospital stay, complications, and recurrence were also documented.

Chronic pain was defined as pain lasting for more than 3 months, according to the International Association for the Study of Pain.^12^ This definition was adopted in our study, and the visual analog scale (VAS) was used to provide an objective assessment. Median (interquartile range [IQR]) VAS scores were recorded at postoperative day 1 (VAS1D), 1 month (VAS1M), and 3 months (VAS3M).

The current EHS guidelines^2^**^,^**^13^ provide no precise definition of recurrence. Patients reporting pain or swelling in the inguinal region were evaluated by an experienced radiologist on ultrasonography, along with a physical examination performed by an independent surgeon. Recurrence was defined as the protrusion of intra-abdominal organs into the inguinal canal during a Valsalva maneuver on ultrasonography and the presence of reducible swelling.

In both groups, the principles of the critical view of the myopectineal orifice (CVMO), as described by Daes et al,^14^ were meticulously followed. After a complete dissection of the direct, indirect, and femoral hernia regions, the cord structures were adequately parietalized, and cord lipomas were routinely identified and repositioned above the mesh. Although current guidelines^2^**^,^**^13^ do not recommend routine mesh fixation in all cases, standardized absorbable tackers were applied in both groups to prevent bias in postoperative pain comparison. In accordance with the CVMO recommendations, all fixation points, except for the Cooper’s ligament, were placed above the interspinal line. Three absorbable tacks were used: 1 at the Cooper’s ligament, 1 lateral to the junction of the mesh legs (central tacker), and 1 at the superolateral corner. In the SM group, the central tacker was deemed necessary to close the internal defect created by the mesh incision and to prevent recurrence through this potential weak point. To ensure standardization and avoid bias in pain-related outcomes, the same fixation pattern was applied in the NSM group. Additionally, fixation was carefully avoided in the anatomical areas defined as “the triangle of pain” and “triangle of doom,” where major neurovascular structures are at risk. This strategy aimed to minimize the risk of injury to the ilioinguinal, genitofemoral, and lateral femoral cutaneous nerves, and iliac vessels. This practice was in accordance with recent anatomical recommendations of Claus et al,^15^ who emphasized that critical neurovascular structures, including branches of the ilioinguinal, genitofemoral, and lateral femoral cutaneous nerves, may extend as far as 2 cm above the iliopubic tract. Therefore, they strongly advise avoiding any mesh fixation within or near the boundaries of the triangle of pain to prevent inadvertent nerve entrapment or injury.

**FIGURE 2:**
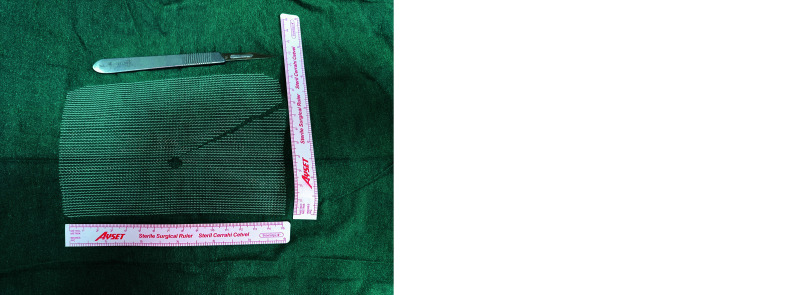
Mesh preparation for a right inguinal hernia; A – nonslit mesh; B – slit mesh

**FIGURE 3:**
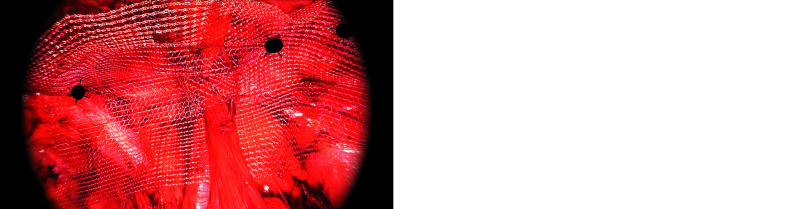
Example caption for this imageSpread of the mesh for a right inguinal hernia; A – nonslit mesh; B – slit mesh

In both groups, a 10 cm × 15 cm polypropylene mesh was used. In the NSM group, the mesh was placed flat without a slit (FIGURE 2A). In the SM group, an oblique lateral slit was created (FIGURE 2B), and the lateral leg of the mesh was passed beneath the vas deferens and gonadal vessels to cover the pubic symphysis medially and the psoas muscle laterally. In the female patients, the round ligament was preserved and treated as the anatomical equivalent of the vas deferens. In the SM group, the lateral leg of the mesh was passed beneath the round ligament using the same technique as in the male patients. During the passage of the lateral mesh limb beneath the cord structures, particular attention was paid to avoid injury to the genital branch of the genitofemoral nerve. Considering the known anatomic variations, including high division of this nerve, the dissection was performed cautiously below the gonadal vessels to minimize the risk of neurovascular damage. The same tacker configuration was used in both groups, as illustrated in FIGURE 3A and 3B, respectively.

### Statistical analysis

Descriptive statistics were presented as numbers and percentages for categorical variables, and as mean (SD) and median (IQR) for continuous variables. The Shapiro–Wilk test was used to assess the normality of continuous data. When parametric assumptions were met, the *t* test was used to compare 2 independent groups; otherwise, the Mann–Whitney test was applied. The χ^2 ^test was used for comparisons of categorical variables. All statistical analyses were performed using IBM SPSS Statistics package version 22 (IBM Corp., Armonk, New York, United States). A *P* value below 0.05 was considered significant.

## RESULTS

A total of 155 patients (145 men and 10 women) underwent laparoscopic TEP hernia repair. Of these, 80 (51.6%) were treated with the SM technique, and 75 (48.4%), with the NSM technique. Mean (SD) age of the cohort was 46.97 (13.96) years. Median (IQR) hernia defect diameter measured on ultrasonography was 15 (13–20) mm. The distribution of hernia location and type is summarized in TABLE 1. Additionally, TABLE 1 includes the EHS classification based on the defect size. The majority of patients had class 1 (53.5%) or class 2 hernias (43.2%), while only a small proportion had large defects classified as class 3 (3.2%). The distribution of EHS classifications was similar between the SM and NSM groups (*P* = 0.27). There were no differences in demographic or hernia-related characteristics between the groups.

**TABLE 1:** Demographic and hernia characteristics

Parameter		Overall (n = 155)	SM (n = 80)	NSM (n = 75)	P value
Sex	Men	145 (93.5)	76 (95)	69 (92)	0.52ª
	Women	10 (6.5)	4 (5)	6 (8)	
Age, y, mean (SD)		46.97 (13.96)	47.46 (13.91)	46.45 (14.09)	0.66ᵇ
Location	Right groin	74 (47.7)	41 (51.2)	33 (44)	0.42ª
	Left groin	81 (52.3)	39 (48.8)	42 (56)	
Hernia type	Indirect	97 (62.6)	49 (61.2)	48 (64)	0.33ª
	Direct	42 (27.1)	20 (25)	22 (29.3)	
	Pantaloon	16 (10.3)	11 (13.8)	5 (6.7)	
Preoperative defect diameter, mm, median (IQR)		15 (13–20)	15 (12–20)	15.5 (15–20)	0.38^c^
EHS class	1 (≤1.5 cm)	83 (53.5)	45 (56.3)	38 (50.7)	0.27ª
	2 (1.5–3 cm)	67 (43.2)	31 (38.8)	36 (48)	
	3 (>3 cm)	5 (3.2)	4 (5)	1 (1.3)	

Data are presented as numbers and percentages unless indicated otherwise.

a. χ2 test

b. t test

c. Mann–Whitney test

Abbreviations: EHS, European Hernia Society; IQR, interquartile range; others, see FIGURE 1

Median (IQR) operation time was 43 (35–50) minutes, with a significantly longer duration in the SM group than the NSM group (46.5 [40–55] vs 38 [30–45] min; *P* <⁠0.001). Median (IQR) length of hospital stay was 1 (1–1) day in both groups. Mean (SD) follow-up lasted for 23.78 (2.76) months, with 23.65 (2.81) months in the SM group and 23.91 (2.71) months in the NSM group. Drain placement was required in 7 patients (4.5%), and conversion to open surgery was necessary in 3 patients (1.9%). There were no intergroup differences in terms of drain placement and conversion (*P* = 0.12; *P* = 0.6, respectively), as shown in TABLE 2.

Intraoperative peritoneal breaches with pneumoperitoneum may occasionally occur due to peritoneal traction; such events are typically managed by decompressing the abdomen via a Veress needle inserted at the Palmer’s point (left upper quadrant). However, in all 3 patients who required conversion in this study, the decision was not related to pneumoperitoneum, but was made at the request of the anesthesia team due to ventilation-related respiratory compromise during the procedure.

Recurrence was observed in 9 patients (5.8%): 6 (7.5%) in the SM group and 3 (4%) in the NSM group. Although the difference was insignificant (*P* = 0.49), recurrence was more frequent in the SM group (odds ratio [OR], 1.95; 95% CI, 0.47–8.08). The most common postoperative complications were seroma in 14 patients (9%), hematoma in 4 individuals (2.6%), and surgical site infection in 1 participant (0.6%). There were no differences between the groups regarding complications (*P* = 0.54). Median (IQR) VAS score was 4 (2–6) at VAS1D, 0 (0–2) at VAS1M, and 0 (0–0) at VAS3M. No differences in the VAS score were found between the groups. The comparative data for recurrence and complications are presented in TABLE 2. After a more in-depth analysis of the recurrence cases, we found that the majority of these patients were men (8 men and 1 woman). No relevant intergroup differences were observed in terms of hernia side (right, n = 5; left, n = 4) or hernia type (indirect, direct, or pantaloon). Most recurrences occurred in the EHS class 1 or 2 patients, and no recurrence was observed in the individuals with class 3 hernias. A detailed breakdown of recurrence cases is provided to offer clinical context for the overall comparison between the 2 techniques.

## DISCUSSION

The SM is a technical modification designed to optimize mesh placement to more closely conform to anatomic structures and to reduce both recurrence and CPIP rates. This concept was first introduced by Korman et al^16^ In that study, no recurrences were reported, while a higher incidence of CPIP was observed in the SM group.

In our study, there were differences between the groups in terms of demographic and clinical characteristics. The homogeneous distribution of baseline variables strengthens the reliability of the results.

Several factors have been identified in the literature as contributing to recurrence, including limited surgical experience, inadequate dissection, inappropriate mesh size, and insufficient mesh coverage.^2^**^,^**^5^**^,^**^17^ In this study, all procedures were performed by experienced surgeons, and a standard 10 cm × 15 cm polypropylene mesh was used in every case. Therefore, the potential influence of surgical experience and mesh size on recurrence was mitigated. Advocates of the SM technique argue that routing the mesh beneath the vas deferens provides an additional anchoring point and may help prevent cranial migration.^3^**^,^**^16^ This rationale is partly based on the hypothesis that the lower edge of the mesh may migrate over time due to physical movement in the groin region. In this context, SM is believed to offer improved mechanical stability by anchoring the mesh beneath the vas deferens and gonadal vessels. However, this concept remains a subject of clinical discussion and has not yet been clearly validated by high-level evidence. In contrast, opponents argue that creating a slit in the mesh could compromise its structural integrity and potentially increase the risk of recurrence.^5^**^,^**^6^**^,^**^9^ Although our cohort did not include any patients with large lateral hernias (L3 according to the EHS classification), the concern regarding insufficient support near the deep ring in such cases is valid. Central tacker placement may be inadequate for securing the mesh in L3 defects, and this could theoretically increase the risk of recurrence. Therefore, we believe that SM should be used with caution in patients with large lateral hernias, and alternative fixation strategies may be warranted.

**TABLE 2:** Comparison of surgical outcomes of the slit mesh and nonslit mesh techniques

Parameter		Overall (n = 155)	SM (n = 80)	NSM (n = 75)	P value
Operation time, min		43 (35–50)	46.5 (40–55)	38 (30–45)	<0.001ᶜ
Length of hospital stay, d		1 (1–1)	1 (1–1)	1 (1–1)	0.17ᶜ
Follow-up, mo		23.78 (2.76)	23.65 (2.81)	23.91 (2.71)	0.55ᵇ
Need for drainage	No	148 (95.5)	74 (92.5)	74 (98.7)	0.12ª
	Yes	7 (4.5)	6 (7.5)	1 (1.3)	
Need for conversion	No	152 (98.1)	78 (97.5)	74 (98.7)	0.6ª
	Yes	3 (1.9)	2 (2.5)	1 (1.3)	
Recurrence	No	146 (94.2)	74 (92.5)	72 (96)	0.5ª
	Yes	9 (5.8)	6 (7.5)	3 (4)	
Postoperative complications	None	136 (87.7)	69 (86.3)	67 (89.3)	0.54ª
	Seroma	14 (9)	8 (10)	6 (8)	
	Hematoma	4 (2.6)	3 (3.8)	1 (1.3)	
	SSI	1 (0.6)	0	1 (1.3)	
VAS1D		4 (2–6)	4 (2–6)	4 (1–5)	0.16ᶜ
VAS1M		0 (0–2)	1 (0–2)	0 (0–2)	0.65ᶜ
VAS3M		0 (0–0)	0 (0–0)	0 (0–1)	0.12^b^

Data are presented as numbers and percentages or median (interquartile range) unless indicated otherwise.

a. Mann–Whitney test

b. t test

c. χ2 test

Abbreviations: SSI, surgical site infection; VAS1D, visual analog scale score on postoperative day 1; VAS1M, visual analog scale score on postoperative month 1; VAS3M, visual analog scale score on postoperative month 3; others, see FIGURE 1

In 2011, Domniz et al^3^ reported recurrence rates of 0.6% in the SM group and 5.9% in the NSM group, suggesting a 10-fold higher recurrence with the NSM technique. However, this study has been strongly criticized by other researchers.^6^**^,^**^8^**^,^**^10^ For example, Koeckerling et al^6^ pointed out that the current guidelines approach does not comprehensively address the SM technique. They also suggested that, in theory, the creation of a slit in the mesh could be associated with higher recurrence. In addition, outcomes may be affected by surgeon experience, particularly during the early stages of the learning curve.

Several studies^7^^,^^8^^,^^9^^,^^10^ have reported no differences in recurrence rates between SM and NSM groups. In a recent retrospective study published in 2024,^4^ 611 patients undergoing laparoscopic IH surgery (SM, n = 353; NSM, n = 258) were analyzed. A multivariate analysis showed that the SM technique was associated with a reduced recurrence rate (OR, 0.228; 95% CI, 0.064–0.809). However, the study had notable limitations, including a short follow-up period (mean [SD], 6.6 months) and variability in mesh types used. Recurrence rates have been shown to correlate directly with follow-up duration. In a comprehensive review, Köckerling et al^18^ emphasized that 42.5% of all hernia recurrences were detected more than 10 years after the initial surgery, highlighting the importance of long-term follow-up in the accurate assessment of recurrence rates. However, a recent meta-analysis of five trials^11^ reported comparable recurrence rates between the SM (10/633) and NSM (12/443) groups. In our study, the overall recurrence rate was 5.8% (9 patients, including 6 in the SM group and 3 in the NSM group). While this difference did not reach significance (*P* = 0.49), recurrence was proportionally higher in the SM group (OR, 1.95; 95% CI, 0.47–8.08). Although the standard 10 cm × 15 cm mesh size was used in all cases, no recurrences were observed among the 5 patients with large (class 3) hernias, including both direct (n = 2) and indirect (n = 3) types. While this does not contradict the current guidelines^2^**^,^**^13^ recommending larger meshes for median M3 hernias, it may suggest that adequate surgical technique and mesh fixation can play a role in minimizing recurrence even in such cases. However, this observation should be interpreted with caution due to the limited sample size. We hypothesize that this finding may be attributed to an internal defect resulting from the slit in the mesh, which may facilitate peritoneal protrusion. Adequate coverage of the myopectineal orifice is essential, as defects that are too wide or too narrow have been associated with recurrence and other postoperative complications.

In a randomized prospective study published in 2023,^19^ 132 men undergoing minimally-invasive IH repair were evaluated to identify risk factors associated with acute postoperative pain. The patients were categorized into mild (VAS, 0–2) and severe (VAS, 3–10) pain groups based on VAS scores recorded 3 hours after surgery. The analysis identified longer symptom duration (>1 year), smoking status, and undergoing transabdominal preperitoneal (TAPP) hernia repair as significant predictors of moderate to severe acute pain. However, the inclusion of both TEP and TAPP procedures within the same study cohort may have influenced the reported outcomes. As these are distinct surgical techniques with different anatomical and technical considerations, we believe that analyzing them separately would strengthen the validity and interpretability of the results, particularly in the context of postoperative pain assessment. In contrast, all patients in our study underwent standard TEP repair, using the same type of mesh and identical tacker placement. This approach enabled us to focus solely on comparing the effects of SM and NSM techniques on surgical outcomes.

Numerous studies have been conducted on mesh selection in IH repair; however, no particular type of mesh has been conclusively shown to be superior to others.^2^**^,^**^13^**^,^**^17^**^,^**^20^ In a randomized controlled trial published in 2022, Zamkowski et al^20^ compared a ProGrip self-fixating mesh (Covidien, Mansfield, Massachusetts, United States) with a standard lightweight macroporous polypropylene mesh in the Lichtenstein procedure. Although the use of the self-fixating mesh resulted in a significantly shorter operative time, it did not demonstrate any advantage in reducing postoperative pain, either in the early or late follow-up periods. In fact, pain severity within the first 30 postoperative days was significantly higher in the self-fixating mesh group. Based on these findings and considering cost-effectiveness, we decided to use a conventional polypropylene mesh in our study.

In the series by Domniz et al,^3^ postoperative groin pain was more frequently reported in the SM group following bilateral IH repair. However, most studies found no significant difference in postoperative groin pain rates between the SM and NSM groups.^3^**^,^**^8^**^,^**^10^**^,^**^11^ In the aforementioned meta-analysis, 4 trials involving a total of 1036 patients reported on postoperative pain. No difference was observed between the SM and NSM group (CPIP rate, 4.4% vs 7.5%, respectively; *P* = 0.99).^11^ Another meta-analysis^21^ reported a 10% incidence of moderate-to-severe CPIP following the TEP technique, with 25% of these surgeries resulting in limitations in daily activities. Identified risk factors for CPIP included preoperative pain, female sex, younger age, nonretired occupational status, psychiatric disorders, recurrent hernia, open surgical approach, and nonmesh repair techniques. Most of these studies assessed the presence of pain without using standardized scoring systems. In our study, we used the VAS to objectively assess groin pain. In the study by Yildirim et al,^7^ median VAS1D score was considerably higher in the SM group, whereas no differences were reported for VAS3M and VAS6M. In contrast, we found no significant differences in any of the median VAS scores (*P* = 0.16; *P* = 0.65; *P* = 0.12 for VAS1D, VAS3M, and VAS6M, respectively). We hypothesize that effective prevention of postoperative pain is closely related to the surgeon’s knowledge of neuroanatomy and careful avoidance of tackers or sutures near the neural structures. Although current evidence is limited, non-absorbable tackers have been suggested to be associated with increased postoperative pain. For this reason, we used absorbable tackers.

In our study, operation time was markedly longer in the SM group than the NSM group (46.5 vs 38 min; *P* <⁠0.001). This can be explained by the additional step required to create a slit and pass the mesh beneath the spermatic cord, which naturally prolonged the procedure. Interestingly, in the series by Chue et al,^4^ mean (SD) operation time was longer in the NSM group than in the SM group (76.7 [30.1] vs 64.6 [29] min). In both groups, surgery times considerably exceeded those typically found in the literature. In a meta-analysis by Demetriou et al,^11^ 3 of the included trials reported data on operative time, and no difference was observed between the groups. In contrast, Yildirim et al^7^ reported a longer operative time in the SM group.

In our study, the most common postoperative complications were seroma (9%) and hematoma (2.6%). In their systematic review, Li et al^22^ reported seroma rates as high as 23.8%. Various techniques have been suggested to reduce seroma formation, including suturing the transverse fascia to the Cooper’s ligament, strangulation of the fascia using an endoloop, closure of the defect with barbed sutures, placement of a drain, and fixation of the distal hernia sac using fibrin glue or sutures.^22^ In a meta-analysis by Demetriou et al,^11^ 3 studies reported on seroma formation, with no difference between the groups (6.9% in the SM group vs 7.5% in the NSM group). In our study, the overall seroma rate was 9% (8 patients in the SM group and 6 in the NSM group), with no intergourp differences. In M2 and larger hernias, the transversalis fascia was retracted and tacked to the Cooper’s ligament in order to minimize seroma formation. This maneuver is believed to contribute to lower postoperative seroma rates.

However, these findings should be interpreted with caution, considering several limitations. First, this was a single-center, retrospective study, and therefore subject to information and recall bias inherent to the study design, despite meticulous and protocol-driven documentation. In addition, selection bias may have been introduced, as the choice between the slit and nonslit mesh was left at the discretion of the surgeon, and the underlying rationale for each decision was not standardized. Although the average follow-up time was acceptable, the findings should be validated in a larger patient cohort. The study also has several strengths, including the use of a standardized mesh size, identical tacker application sites across the groups, well-balanced baseline characteristics, and follow-up evaluations conducted by an independent clinician, not involved in the surgical procedures.

## CONCLUSIONS

In this study, the use of SM was associated with longer operation time, without a noteworthy reduction in recurrence or postoperative pain rates. Although not significant, recurrence was proportionally more frequent in the SM group. Further research with larger sample sizes and extended follow-up periods is warranted to better understand the clinical implications of SM placement.

## JOURNAL INFORMATION

Videosurgery and Other Miniinvasive Techniques is an official journal of the Videosurgery Foundation.
